# Knowledge, Attitudes and Practices toward Hepatitis B Virus Infection among Students of Medicine in Vietnam

**DOI:** 10.3390/ijerph18137081

**Published:** 2021-07-02

**Authors:** Thi Thuy Linh Nguyen, Thi Thanh Hang Pham, Samuel So, Thi Hai Van Hoang, Thi To Uyen Nguyen, Thanh Binh Ngo, Minh Phuong Nguyen, Quang Hung Thai, Ngoc Khoi Nguyen, Thi Quynh Anh Le Ho, Quang Phuc Tran, Minh Khue Pham

**Affiliations:** 1 Faculty of Public Health, Haiphong University of Medicine and Pharmacy, Haiphong 04212, Vietnam; ntthuylinh@hpmu.edu.vn (T.T.L.N.); tqphuc@hpmu.edu.vn (Q.P.T.); 2 Asian Liver Center, Department of Surgery, Stanford University School of Medicine, Palo Alto, CA 94304, USA; hpham3@stanford.edu (T.T.H.P.); samso@stanford.edu (S.S.); 3 Department of Global Health, Hanoi Medical University, Hanoi 11521, Vietnam; hoangthihaivan@hmu.edu.vn; 4 Faculty of Public Health, Thai Nguyen University of Medicine and Pharmacy, Thai Nguyen 24117, Vietnam; ngtouyen75@gmail.com; 5 Department of Oto-Rhino-Laryngology, Thai Binh University of Medicine and Pharmacy, Thai Binh 06118, Vietnam; ngothanhbinh@tbump.edu.vn; 6 Department of Pediatrics, Can Tho University of Medicine and Pharmacy, Can Tho 94117, Vietnam; nmphuong@ctump.edu.vn; 7 Department of Public Health, Faculty of Medicine and Pharmacy, Tay Nguyen University, Dak Lak 63000, Vietnam; tqhung@ttn.edu.vn; 8 Department of Clinical Pharmacy, University of Medicine and Pharmacy at Ho Chi Minh City, Ho Chi Minh City 71006, Vietnam; nnkhoi@ump.edu.vn; 9 Family Medicine Center, Hue University of Medicine and Pharmacy, Hue University, Hue 49000, Vietnam; lhtqanh@huemed-univ.edu.vn

**Keywords:** HBV, knowledge, medical students

## Abstract

Background: Building capacity in hepatitis B virus prevention and management for medical students and health professionals is one of the pillars of the national viral hepatitis control strategy. Methods: A cross-sectional study was conducted at eight medical universities from the northern, central and southern regions of the country between May and November 2020 using a systematic random sampling technique. Results: Among 2000 participants, 84.2% reported they had been tested for hepatitis B and 83.9% had received the hepatitis B vaccine. The mean knowledge, attitude, practice score was 40.2 out of 54 (74.4%) with only 19.9% of the study participants obtaining a good score. In multivariate analysis, fifth year students, students from central universities, students who had tested positive for hepatitis B and students who had received hepatitis B vaccine or had encountered patients with chronic hepatitis B had significantly higher knowledge score (*p* < 0.05). The study showed lack of trust in the hepatitis B vaccine safety and lack of confidence in providing counselling, testing and management of patients with chronic hepatitis B. Conclusion: Findings from our research emphasized an immediate need to improve the medical schools’ training curriculum in Vietnam to enable students’ readiness in hepatitis B prevention and management.

## 1. Introduction

Vietnam has a high burden of viral hepatitis, with hepatitis B and C as the major causes of liver cirrhosis, liver cancer and liver-related deaths. The country ranked sixth in the incidence of liver cancer and had the third highest rate of death from liver cancer in the world in 2018 [1]. A recent estimation and projection by the Ministry of Health, Center for Disease Analysis and the World Health Organization (WHO) reports that 1 out of 11 people in Vietnam is living with chronic hepatitis B (CHB) or C infection. Without intensified interventions, complications and liver-related deaths due to viral hepatitis will continue to rise [2]. The menace of this disease can be prevented to a great extent by newborn vaccination program, increased awareness and good practice of HBV disease. Hepatitis B vaccine is highly effective in preventing both vertically and horizontally acquired chronic HBV infection [3,4,5]. Hepatitis B vaccine is available in healthcare system in Vietnam including health centers and clinics with monovalent vaccine since 2003 and pentavalent DPT-Hib-Hepatitis B vaccine for children since 2010 [6].

The rate of hepatitis B virus infection among health professionals is about 2 to 10 times higher than that of the general population due to occupational exposures [7]. It is estimated needle injury causes about 66,000 annual HBV infections worldwide [8]. WHO has recommended universal vaccination for all health care workers to protect them from HBV infection [9]. In Vietnam, HBV testing, vaccination and post-vaccination screening to evaluate immunity status are recommended, but not required, for medical professionals.

With HBV infection remaining a major public health problem in Vietnam, capacity building of health care workers is one of the main strategies in the national action plan for hepatitis control [10]. Medical students are part of healthcare workers system. They are at higher risk of exposure than HCWs due to lack of experience to minimize the risk of infection in the healthcare setting and professional skills about screening, monitoring and management of CHB patients. In medical schools, training on hepatitis B prevention and control is integrated into a series of modules, including the infectious diseases and the epidemiology modules for students during the fifth year. Competency in the five core interventions (prevention, testing, monitoring, management and treatment) is important to reduce the burden of viral hepatitis. Worldwide, a number of studies [9,10,11,12,13,14] have assessed knowledge, attitude and behavior of medical students regarding hepatitis B control and highlighted the need for improvement. Therefore, there appears to be a need to improve the training curriculum for medical students [11,12,13,14,15,16]. This study aimed to evaluate the knowledge, attitudes and practices of medical students in medical universities in Vietnam about HBV prevention and control to identify opportunities for improving the capacity of medical students to eliminate new HBV infections and reduce the burden of CHB.

## 2. Materials and Methods

This cross-sectional study was conducted from May to November 2020 at eight representative medical universities located in the northern, central and southern regions of Vietnam (Ha Noi Medical University, Hai Phong University of Medicine and Pharmacy, Ho Chi Minh City University of Medicine and Pharmacy, Hue Medicine University, Can Tho University of Medicine and Pharmacy, Thai Nguyen University of Medicine and Pharmacy, Thai Binh University of Medicine and Pharmacy, Tay Nguyen University’s Faculty of Medicine). Each university reportedly had about 300–500 students in their 5th or 6th year. 2000 of them (250 students at each university) were recruited using systematic random sampling technique. All students participating in this study had completed the infectious diseases and the epidemiology training courses.

### 2.1. Questionnaire

A self-administered questionnaire was developed based on a previously validated tool used to evaluate HBV knowledge, attitude and practice of health professionals in Vietnam [10]. Before distribution, the predesigned questionnaire was tested on a random sample of students (*n* = 20) to ensure understandability and clarity of the questions. There were 43 questions on HBV knowledge, 8 questions on HBV attitudes and 3 questions on practices (See Supplementary File S1).

### 2.2. Definitions for Scoring Knowledge, Attitudes and Practices (KAP)

The following operational definitions were used in this study. Each correct answer was given 1 point. Each incorrect answer received 0 point. The score of a participant was considered good if the individual answered >70% of the questions in each KAP category correctly. The score of participants who correctly answered less than 70% of the questions in each category was considered poor. 

### 2.3. Data Collection and Analysis

Data was entered into EpiData 3.1 (EpiData Association, Odense DK5230, Denmark) then imported and analyzed using STATA v14.0 (Stata Corp, College Station, TX, USA). The 95% confidence intervals (CI) of ratios were calculated using the Clopper–Pearson method. Students’ KAP-related factors about hepatitis virus were determined by the single-variable logistic regression method. Factors related to the students’ KAP variables with *p* < 0.20 in the univariate analysis were included in the multivariate logistic regression model. The model’s fitness was assessed using the link test and the Hosmer–Lemeshow test. All tests were two-tailed and *p* < 0.05 was considered statistically significant.

### 2.4. Ethical Consideration

The study was approved by the Institutional Ethics Committee of Haiphong University of Medicine and Pharmacy (No. 113/HDDD on 5 May 2020). Written informed consent was obtained from all participants prior to the survey. No personal identifying information was collected to ensure confidentiality for participants.

## 3. Results

### 3.1. Demographics of Survey Participants

All 2000 students who were invited to participate in the study returned complete questionnaires, resulting in 100% response rate. Characteristics of the study participants are presented in Table 1. About 54.4% of them were female and 45.5% were male, and their mean age was 23.7 ± 0.9 years (ranging from 21 to 30 years). About 84.2% self-reported that they had been tested for hepatitis B and 83.9% had received the hepatitis B vaccine. Among the 1685 participants who were tested for hepatitis B, 83 (4.9%) reported they were positive for HBsAg. Majority of the participants (94.3%) had previously encountered hepatitis B patients when practicing at teaching hospitals.

### 3.2. Knowledge about Hepatitis B Prevention, Screening and Treatment

A majority of the study participants (88.4%) were aware that CHB can cause liver cirrhosis, liver failure, liver cancer or premature death. However, only 20.7% knew about the high risks for premature death without monitoring and treatment. About 41.4% knew the estimated prevalence rate of CHB in Vietnam. Knowledge about the risk of developing chronic infection related to the age of initial exposure to the hepatitis B virus was poor. Only 44.5% were aware that newborns who become infected with HBV are at the highest risk of developing CHB, and only 35.2% knew that mother-to-child transmission at birth is the most common cause of CHB in Vietnam (Table 2).

**Figure 1:**
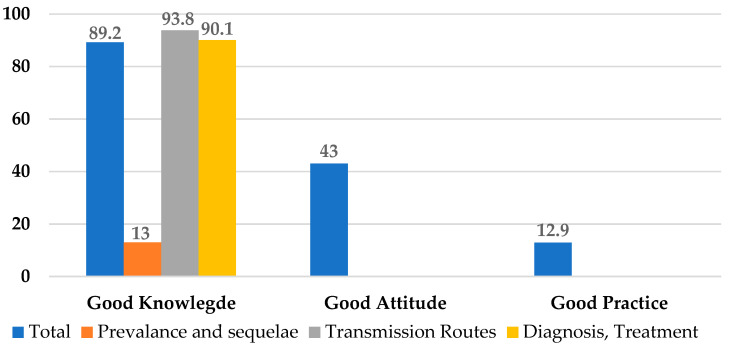
Good KAP about hepatitis B.

As shown in Table 2 and Figure 1, the surveyed participants had good knowledge on the transmission routes of HBV. Most of them provided correct answers that HBV can be transmitted through mother-to-child at birth (96.7%), unprotected sex (96.8%), blood transfusion (96.7%), but not by sneezing or coughing (94.9%) or handshake (97.5%). However, 13.5% still wrongly believed that HBV could be transmitted through sharing utensils and foods with HBV infected persons and that cleaning and cooking foods thoroughly could prevent HBV transmission.

Most of the participants (97.5%) recognized hepatitis B vaccination could prevent HBV infection. About 95.7% knew vaccinating newborns at birth could prevent mother-to-child transmission and that the first dose of hepatitis B vaccine should be given within 24 h after birth (91%). However, fewer participants (73.8%) knew that giving the first dose of hepatitis B vaccine and hepatitis B immune globulin (HBIG) within 12 h of birth and completing the vaccine series is the most effective way to protect infants born to HBsAg positive mothers from becoming infected. 

Knowledge about injection safety was assessed by three questions (Table 2). Only about half of the respondents (56.6%) knew they should not recap the needle with two hands to prevent needle stick injury. Only 70.5% knew they should dispose used needle and syringe into a sharp-proof container immediately without recapping the needle.

Most of the participants knew that HBV screening is recommended for pregnant women (95.3%), HIV patients (95.1%), men who have sex with men (93.8) and family members of hepatitis B infected patients (95.6%). Majority of them (88.9%) knew the hepatitis B surface antigen (HBsAg) test is used to confirm a patient has CHB. Fewer participants (71.7%) knew that the hepatitis B surface antibody (anti-HBs) test is the test used to identify if a patient has immunity to hepatitis B. However, only 6.2% provided the correct response about when to test for HBsAg in infants born to HBsAg positive mothers to assess whether they have become infected despite being vaccinated.

The participants’ knowledge about hepatitis B management and treatment was inadequate. Only 33.5% knew that most patients with CHB often present no symptoms. About 69.4% were aware that there is no cure for CHB but there are effective medications to manage and control the disease. About 30.1% wrongly believed that all CHB patients need to receive antiviral treatment. About a third (33.6%) was not aware that treatment of CHB with NAs (nucleotide or nucleoside analogues) is long-term and potentially lifelong.

### 3.3. Attitudes about Hepatitis B

While a majority of study participants (95.7%) believed it was necessary to vaccinate newborns with the hepatitis B vaccine at birth, there was a lack of trust in the safety of the hepatitis B vaccine with 61% being confident that the hepatitis B vaccine is safe. About two thirds (67.9%) were confident in counselling patients on HBV prevention. Similarly, about 63.9% of the study participants were confident in ordering laboratory tests to diagnose CHB and 61.5% confident in ordering laboratory tests to monitor patients with CHB. Only 27.5% of the study participants were confident in prescribing medications to treat CHB patients (Table 3).

Hepatitis B stigma remained common among the study participants. About 59.3% expressed concerns about casual contact or working in the same office, and 65.2% expressed concerns about eating with or sharing food with a person with CHB (Table 3). 

### 3.4. HBV Preventive Practices

Among the surveyed students, only 24.9% responded that they sought to receive hepatitis B testing and 20.1% sought to receive the hepatitis B vaccine before entering practicum experiences at teaching hospitals. About 68.7% reported that they consistently wore gloves when administrating injection or performing medical procedures at teaching facilities. There was no significant difference in practices across the participating schools (Table 4).

### 3.5. Knowledge, Attitudes and Practice Scores Associated Factors

Mean scores for knowledge, attitudes and practices are shown in Table 5. The mean score for HBV knowledge was 34.1 out of 43 (79.3%). The highest knowledge sub-categories mean scores were 5.7 out of 6 (95.0%) and 10.9 out of 13 (83.8%) in HBV transmission routes and prevention, respectively. However, the knowledge sub-categories mean scores were only 2.3 out of 5 (46.0%) and 7.9 out of 19 (41.6%) for CHB prevalence and sequelae, and for diagnosis and treatment, respectively. The mean score for HBV attitudes was 5 out of 8 (62.9%), and the mean score for HBV preventive practices was only 1.1 out of 3 (36.7%).

Multivariate analysis showed that female students (AOR 1.76; 95%CI: 1.28–2.41); fifth year students (AOR 2.95; 95%CI: 2.02–4.31); studying at Central universities (AOR 2.08; 95%CI: 1.41–3.07); having received hepatitis B vaccine before (AOR 2.39 95%CI: 1.58–3.61); and having encountered a hepatitis B patient before (AOR 0.19; 95%CI: 0.12–0.28) were significantly associated with better knowledge score (Table 6).

## 4. Discussion

This study was the first multi-site survey enrolling medical students in their final two years of training across eight medical universities located in the northern, central and southern regions of Vietnam to assess HBV knowledge, attitudes and practices. As such, it provides the first national data on HBV KAP and the situation of HBV testing and vaccination in medical students in the country. Overall, the percentages of participants who reported receiving HBV testing and vaccination before entering practicum at teaching hospitals were 24.4% and 20.1%, respectively. At the time of this study, about 84.2% of the study participants have been tested for hepatitis B. The percentage of students having received the hepatitis B vaccine was 83.9%, higher than previously reported among health care workers (HCWs) (68.8%) in Vietnam [10] and higher than was estimated by WHO for its 14 geographical regions (18%–77%) [8]. In 2006, following the World Health Organization (WHO) recommendation, Vietnam officially implemented the first dose of hepatitis B vaccination for the infants within the first 24 hours after birth [17]. Since most of the medical students were born before 2006, they likely have not received timely birth dose within 24 h after birth. The Vietnam national guidelines for hepatitis B diagnosis and treatment recommends hepatitis B vaccination for all medical professionals [18], and ideally immunity should be achieved before they enter practicum at teaching hospitals or clinics where they could be exposed to infected blood or bodily fluids. To achieve universal hepatitis B vaccine coverage for all medical students, medical schools need to implement initiatives to evaluate students’ immunity status and provide vaccines to unvaccinated individuals. 

Overall, the mean KAP score was 40.2 (74.4% of the maximum score), indicating a fairly good knowledge level of the study participants. However, only about one fifth of them (19.9%) obtained a good KAP score, which was defined as providing correct answers to at least 70% of the questions. There was a difference in KAP scores between the school years, with students in the fifth year having higher scores than those in the sixth year. It is paradoxical because students in their sixth year have gained more training and experience through clinical placements. However, it is possible that because the fifth year students had just finished training courses on infectious diseases and epidemiology, they had more recent knowledge acquisition about HBV and the vaccine. Similar findings were reported in a study from Saudi Arabia in which medical students in their third year, who had completed the training course on community medicine prior to participating in the survey, had better knowledge scores than students in their fourth and fifth year [19]. In this study, KAP of students also varied by regions, with students in the central regions having higher knowledge scores than in the North and the South. 

A thorough knowledge about HBV transmission routes, preventive measures, risks and consequences of CHB is important to enable medical students to take preventive measures to protect themselves from getting infected through occupational exposures during their clinical postings as well as to help to educate the public about HBV. This study shows that while the surveyed students had good knowledge about HBV transmission routes, preventive measures and consequences of CHB (>86.5%), they were less knowledgeable about the HBV burden in Vietnam and the relation between age of infection and the development of CHB. Only 39.2% knew about the prevalence of CHB in Vietnam and 35.2% were aware that mother-to-child transmission at birth is the most common cause of CHB in Vietnam. This is consistent with findings from a previous survey in HCWs in Vietnam in which only 41.4% knew about HBV prevalence and 39.5% were aware of the most common cause of CHB. In this study, the percentage of participants knowing about the high risk of developing CHB when infected at birth was 44.5%, compared with 24.5% of the HCWs in the previous study. In this study, the percentage of participants who incorrectly answered that HBV is transmitted through food was 13.5%, lower than in previous studies in HCWs and medical students [10,13,20]. The findings suggest that the training curriculum for medical students should also put emphasis to epidemiological aspects, such as prevalence, burden and risk factors of diseases, in addition to transmission routes and prevention measures.

Medical students are known to be at greater risk for HBV infection during their professional training due to lack of experience and professional skills [14,21]. In this study, knowledge of the study participants about injection safety was lacking; almost a half (43.4%) did not know they should not recap the needle with two hands to avoid needle stick injury and 29.5% were not aware they should dispose of the used needle and syringe into a sharp-proof container immediately without recapping the needle. The findings are in line with previous studies, which have consistently shown that knowledge and practice about injection safety in medical and health science students were inadequate. Those studies also reported that needle-stick injury prevalence varied from 42.8% to 71.1% among medical students [8,10,15,16,21,22,23,24,25]. However, this study neither evaluated the participants’ injection technique nor their experience with needle stick injury at teaching hospitals. A recent study in Laos demonstrated that 86% of students in the healthcare professions scored poorly on an assessment of knowledge about HBV infection [26]. Current data are lacking on the level of awareness of HBV infection among the general population and healthcare personnel in Southeast Asia [27]. Needless to affirm that today’s medical students will become the future health care providers. Therefore, it is important to protect our future health care workforce from HBV by ensuring each student is aware of the occupational exposure hazards and making sure they are vaccinated before they attend practicum at clinical hospitals.

With as many as one in eight people in Vietnam living with CHB, it is important that primary care physicians have adequate knowledge and skill about who needs to be screened, which diagnostic tests to order and how to monitor and manage patients with CHB. A recent study revealed a serious lack of knowledge about interpretation of hepatitis B test results, CHB symptoms and monitoring and treatment of patients with CHB even among HCWs [10]. The same study showed that few participants had adequate knowledge about recommended tests to monitor liver damage (1.6%) and screen for liver cancer (2.5%) and only 13.7% were aware that most CHB patients are asymptomatic. In our study, while a majority of surveyed students provided correct answers to questions related to diagnostic tests (>70%) and treatment principles for CHB (>90%), only 33.5% were aware that most of the CHB patient are asymptomatic, and 32.5% could name first line medications for CHB treatment. As a result, the surveyed students in this study expressed low confidence in recommending diagnostic tests (63.9%), monitoring tests (61.5%) and treatment options for CHB patients (27.5%).

These results emphasize a critical need to reform medical schools’ training curriculum on screening, diagnosis and management of CHB patients. Therefore, training programs of medical universities in Vietnam should be reviewed and revised according to the need of responding to the high burden diseases such as CHB. Currently, most medical universities in this country are revising their medical curriculum and started implementing competency-based medical training. This health problem should not be neglected. This change will help to provide students with the necessary knowledge, attitude and skills at their graduation.

Globally, WHO aims to reduce new viral hepatitis infections by 90% by 2030 from the baseline data in 2015. With mother-to-child at birth being the primary transmission route of HBV infection in Vietnam, efforts to increase vaccination coverage, especially vaccination of newborns at birth, are crucial. These efforts must include raising awareness about the importance of at-birth and follow-up doses of the hepatitis vaccine and making sure they are widely available and affordable. In our study, only 61.0% of the study participants were confident that the hepatitis B vaccine is safe. This is consistent with findings from previous studies, which reported lack of trust in the safety of the hepatitis B vaccine among HCWs (61.2%) and pregnant women (72.9%) in Vietnam [10]. Some studies have also shown that the decision to delay or deny the vaccine to parents was related to the safety of vaccines [28,29]. However, CHB is preventable by a safe and effective vaccine. Lack of knowledge about the safety and efficacy of the hepatitis B vaccine would potentially impede efforts to improve hepatitis B vaccination coverage. Thus, the training curriculum for medical students should also put emphasis on the hepatitis B vaccine in addition to epidemiological or clinical aspects. In addition, medical students need to be informed and required to be vaccinated upon their admission to medical school or at least before their clinical rotation years, i.e., starting from the third year of medical training.

Stigma and lack of knowledge have been acknowledged as significant barriers to the prevention, diagnosis and treatment of chronic infection [25,30,31,32]. Negative attitudes toward HBV infection and unwillingness to provide care for CHB patients has been well documented in previous studies among medical students and HCWs in Vietnam, Japan, Iran, Saudi Arabia and other countries [10,25,30,31,32,33,34,35]. These studies also pointed out that students’ attitude is positively correlated with their mean knowledge scores [10,36] and the level of willingness is significantly associated with their confidence in protecting themselves against infection. The present study showed that the participants had ambivalent attitudes toward HBV, 59.3% of them being concerned about casual contact and 65.2% concerned about sharing food or eating with a person with CHB. Providing professional education to medical students to increase their knowledge, eliminate misconception and help reduce the stigma and discrimination associated with HBV is an effective way forwards to improve prevention and control of HBV in Vietnam.

It is important to note that our study targeted medical students, who have educational backgrounds and basic medical skills wider than other populations. Results from our survey while assessing the levels of KAP and defining gaps in KAP for medical students, might not be representative for other populations. Based on the KAP gaps identified, we would like to recommend medical universities in Vietnam to develop a competency-based medical training program to help medical students acquire basic medical skills.

The study has limitations, including that data were based on self-reporting of the participants and could not be validated. Similarly, estimation of testing and vaccination coverage as well as seroprevalence of HBsAg were based on the reported responses of the participants with no attempt made to verify them.

## 5. Conclusions

This study highlights inadequate knowledge about HBV, low trust in the hepatitis B vaccine and lack of confidence in giving counselling, diagnosis to patients with CHB and to manage them. Hence, it is highly advocated that medical universities must reform their training curriculum to provide essential knowledge and skills to medical students. In addition, medical schools should implement initiatives to provide HBV testing and vaccination to all students attending medical training to achieve universal coverage.

## Figures and Tables

**Table 1:** Demographics of survey participants (*n* = 2000).

Respondents’ Demographics		*n*	%
Age		Mean: 23.7 (SD 0.9, Range 21–30)	
Gender	Male	910	45.5
	Female	1090	54.4
Medical school location in Vietnam (Region)	North	1000	50.0
	Central	250	12.5
	South	750	37.5
Medical school year	5th year	1250	62.5
	6th (final) year	750	37.5
Have you been tested for hepatitis B?	No	315	15.8
	Yes	1685	84.2
	Positive	83	4.9
	Negative	1581	93.8
	Unknown	21	1.3
Did you receive the hepatitis B vaccine?	Yes	1678	83.9
	No	230	11.5
	Unknown	92	4.6
Have you encountered any CHB patient before?	Yes	1885	94.3
	No	115	5.8

**Table 2:** Knowledge about HBV, prevention and treatment (*n* = 2000).

Questions	Correct Answers	
	*n*	%
Prevalence and Sequelae		
A4. What percentage of the Vietnam population has chronic hepatitis B (CHB)?	783	39.2
A5. How did most people who have CHB in Vietnam get infected?	704	35.2
A6. Which age group is most likely to develop CHB after the initial infection?	890	44.5
A7. What are the consequences of chronic hepatitis B?	1767	88.4
A52. Without proper monitoring and treatment, what is the chance a patient would die of CHB complications?	414	20.7
Transmission Routes		
A8. Can hepatitis B be transmitted through handshake?	1949	97.5
A9. Can hepatitis B be transmitted through unprotected sex?	1936	96.8
A10. Can hepatitis B be transmitted through blood transfusion?	1933	96.7
A11. Can hepatitis B be transmitted through sneezing or coughing?	1898	94.9
A12. Can hepatitis B be transmitted through from mother to child at birth?	1933	96.7
A13. Can hepatitis B be transmitted through sharing food or utensils?	1729	86.5
Prevention		
Prevention Measures		
A14. Can cleaning and cooking food thoroughly prevent HBV transmission?	1501	86.5
A15. Can the hepatitis B vaccine prevent HBV transmission?	1949	97.5
A16. Can HBV transmission be prevented by not reusing or sharing needles/syringes?	1945	97.3
A17. Can HBV transmission be prevented by avoid sharing food/utensils or eating with a person with chronic HBV?	1600	80.0
A18. Can using a condom prevent HBV transmission?	1918	95.9
A19.What is the most effective preventive measure for infants born to mothers with chronic HBsAg?	1475	73.8
A21. Who needs the hepatitis B vaccine?	1881	94.1
A23. Prevention of mother-to-child transmission	1712	85.6
A24. The first dose of hepatitis B vaccine for baby	1823	91.2
A33. Is it necessary to have sharp-proof containers at clinics for disposing of needles and sharp objects?	1910	95.5
What would you do to prevent needle-stick injury?		
A30. Wash hands with soap or disinfectant after each clinical procedure?	1694	84.7
A31. Recap needle with two hands after use and discard immediately in a sharp-proof container	1131	56.6
A32. Do not recap needle and discard immediately in a sharp-proof container	1409	70.5
Diagnosis and Treatment		
Who should be tested for hepatitis B?		
A35. Pregnant women should be tested for hepatitis B	1906	95.3
A36. HIV-infected people should be tested for hepatitis B	1902	95.1
A37. Men who have sex with men (MSM) should be tested for hepatitis B	1875	93.8
A38. Family members of those who have hepatitis B should be tested for hepatitis B	1911	95.6
A56. Serum HBsAg test for identification of patients infected with hepatitis B virus	1777	88.9
A57. What test should be used to identify immunity against the hepatitis B virus?	1433	71.7
Diagnosis		
A40. What is the symptom most patients with chronic hepatitis B present?	669	33.5
A41. What are the criteria for indicating treatment in patients with CHB?	1645	82.3
A55. When should infants born to mothers with CHB be evaluated for HBsAg status?	123	6.2
Treatment		
A42. There is no cure, but there are effective medications to manage and control the disease	1387	69.4
Treatment goal: What are the treatment goals for CHB patients?		
A43. Inhibit the replication of the hepatitis B virus	1843	92.2
A44. Prevent disease progression of disease, particularly liver cirrhosis and liver cancer	1938	96.9
A45. Prevent mother-to-child transmission (MTCT)	1927	96.4
A46. Prevent flare of hepatitis B	1855	92.8
Treatment principles		
A47. Is it true that Nucleos(t)ide Analogs (NAs) are a recommended first-line treatment for CHB?	1349	67.5
A48. Is treatment of CHB with NAs long term, possibly even lifetime?	1528	76.4
A49. Do patients need to strictly adhere to the treatment of CHB?	1923	96.2
A50. Do you think that all patients with chronic HBV need to be treated immediately?	1397	69.9
A51. Should all CHB patients be monitored and tested regardless of treatment status?	1830	91.5

Abbreviation: CHB, chronic hepatitis B; HBV, hepatitis B virus; MSM, men who have sex with men; MTCT, mother-to-child transmission; NAs, Nucleos(t)ide Analogs.

**Table 3:** Attitude about HBV (*n* = 2000).

Questions	Answered Yes	
	*n*	%
A20. Are you confident in counseling patients about prevention of HBV?	1358	67.9
A22. Do you think that the hepatitis B vaccine is safe?	1220	61.0
A25. Do you think it is necessary to vaccinate newborns for hepatitis B at birth?	1914	95.7
A53. Are you confident in ordering laboratory tests to monitor CHB patients?	1229	61.5
A54. Are you confident in prescribing treatment for a patient with chronic hepatitis B?	550	27.5
A58. Are you confident in ordering diagnosis tests for patients with chronic HBV?	1279	63.9
A59. Would you have any concerns having casual contact or working together with a chronic HBV patient in the same office?	1185	59.3
A60. Would you have any concerns sharing food or utensils with a CHB?	1303	65.2

**Table 4:** HBV Preventive Practices (*n* = 2000).

Questions	Answered Yes	
	*n*	%
A28. Did you get the hepatitis B vaccine before entering practicum at teaching hospitals?	402	20.1
A29. Did you get tested for HBV before entering practicum at teaching hospitals?	499	24.9
A34. Do you consistently wear gloves when administrating injections or performing medical procedures to patients?	1373	68.7

**Table 5:** Mean score of knowledge, attitudes and practices (*n* = 2000).

Hepatitis B	Number of Questions	Mean	SD (Range)	Percentage of Maximum Score
**Knowledge**	43	34.1	4.2 (7–41)	79.3%
Prevalence and sequelae	5	2.3	1.1 (0–5)	46.0%
Transmission routes	6	5.7	0.8 (0–6)	95.0%
Prevention	13	10.9	1.7 (2–13)	83.8%
Diagnosis, treatment	19	7.9	1.4 (0–9)	41.6%
**Attitude**	8	5.0	1.8 (0–8)	62.5%
**Practice**	3	1.1	0.9 (0–3)	36.7%

**Table 6:** Analysis of factors associated with knowledge about HBV.

	Good Knowledge (*n* = 1784)	OR (95%CI)	*p* *	AOR (95%CI)	*p* **
Gender (*n* = 2000)					
Male (*n*= 910)	785 (86.3)				
Female (*n* = 1090)	999 (91.7)	1.74 1.31–2.33	<0.001	1.76 1.28–2.41	<0.001
Year of Education (*n* = 2000)					
5th (*n* = 1250)	1151 (92.1)	2.15 1.61–2.86	<0.001	2.95 2.02–4.31	<0.001
6th (*n* = 750)	633 (84.4)				
Region (*n* = 2000)					
North (*n* = 1000)	873 (87.3)				
Middle (*n* = 250)	235 (94.0)	2.27 1.30–3.97	0.004	2.08 1.41–3.07	<0.001
South (*n* = 750)	676 (90.1)	1.33 0.98–1.80	0.07	1.07 0.58–1.96	0.83
Test HBV before (*n* = 2000)					
Yes (*n* = 1685)	1548 (91.9)	3.78 2.78–5.15	<0.001	1.06 0.56–2.01	0.85
No (*n* = 315)	236 (74.9)				
Status of HBV (*n* = 2000)					
Positive (*n* = 115)	94 (81.7)	0.37 0.22–0.61	<0.001	0.60 0.34–1.08	0.09
Negative (*n* = 1610)	1487 (92.4)				
Unknown (*n* = 275)	203 (73.8)	0.23 0.16–0.32	<0.001	0.35 0.18–0.69	0.002
Got HBV vaccine before (*n* = 2000)					
Yes (*n* = 1678)	1551 (92.4)	4.31 3.05–6.09	<0.001	2.39 1.58–3.61	<0.001
No (*n* = 230)	170 (73.9)				
Unknown (*n* = 92)	63 (68.5)	0.77 0.45–1.30	0.33	0.72 0.39–1.29	0.27
Met a hepatitis B patient before (*n* = 2000)					
Yes (*n* = 1885)	330 (17.5)	10.23 6.86–15.3	<0.001	8.09 5.20–12.59	<0.001
No (*n* = 115)	18 (59.1)				

Abbreviation: OR, Odds Ratio; AOR, Adjusted Odds Ratio; CI, Confidence Interval. * Univariate analysis, ** Multiple logistic regression.

## Data Availability

The STATA data used to support the findings of this study are available from the corresponding author upon request.

## Supplementary Materials

The following are available online at https://www.mdpi.com/article/10.3390/ijerph18137081/s1, Supplementary File S1: Questionnaire of the survey.
