# Supplementary material for: Knowledge, Attitudes and Practices toward Hepatitis B Virus Infection among Students of Medicine in Vietnam
Source: Int J Environ Res Public Health. 2021 Jul 2;18(13):7081. doi: 10.3390/ijerph18137081 (PMC8296898; doi:10.3390/ijerph18137081)
Supplement: Supplementary file 1 [file ijerph-18-07081-s001.zip › ijerph-1239524-supplementary.pdf]

Supplementary File S1: Questionnaire of the survey.

MEDICAL STUDENTS SURVEY

Student ID: _________

DEMOGRAPHICS

1. **Current age (years)** : _____________ **2. Gender:** ☐ Male ☐ Female

3. Current University (*please choose ONE*):

☐ Faculty of Medicine and Pharmacy- Tây Nguyên University
☐ Cần Thơ Medicine and Pharmacy University
☐ Hải Phòng Medicine and Pharmacy Univeristy
☐ Huế Medicine and Pharmacy University
☐ Thái Bình Medicine and Pharmacy University
☐ Thái Nguyên Medicine and Pharmacy University
☐ Hồ Chí Minh City Medicine and Pharmacy University
☐ Hà Nội Medicine University

DISEASE BURDEN, TRANSMISION ROUTES AND PREVENTION MEASURES

4. In your opinion, approximately how many percent of Vietnam population has hepatitis B? (*Please check ONE*):

A. 1% (1 in 100 individuals)
B. 5% (1 in 20 individuals)
C. 10% (1 in 10 individuals)
D. 25% (1 in 4 individuals)

5. In your opinion, how did most people who have chronic hepatitis B in Vietnam got infected? (*Please check ONE*):

A. Infected mother to child at birth
B. Unsafe injections
C. Contaminated food or water
D. Unprotected sex

6. A person is most likely to develop chronic hepatitis B infection after the initial infection at? (*Please check ONE*):

A. Newborns
B. Teenagers
C. Middle-age and seniors
D. Age is not a factor

7. In your opinion, HBV can cause which of the followings? (*Please check ONE*):

A. Liver cirrhosis
B. Liver failure
C. Liver cancer
D. Premature death

E. All of the above

Question 8-13: How could HBV be transmitted?

8. **Shaking hands with a person infected with HBV**
   A. True
   B. False
   C. Don’t know
9. **Having unprotected sex with a person infected with HBV**
   A. True
   B. False
   C. Don’t know
10. **Having blood transfusion**
   A. True
   B. False
   C. Don’t know
11. **Sneezing or coughing**
   A. True
   B. False
   C. Don’t know
12. **From mother to her child at birth**
   A. True
   B. False
   C. Don’t know
13. **Eating with or sharing food and utensils with a person with HBV**
   A. True
   B. False
   C. Don’t know

Questions 14-19: What can prevent hepatitis B transmission?

14. **Clean and cook food thoroughly**
   A. True
   B. False
   C. Don’t know
15. **Provide hepatitis B vaccination to persons with no immunity**
   A. True
   B. False
   C. Don’t know
16. **Do not reuse or share needles/syringes**
   A. True
   B. False
   C. Don’t know
17. **Avoid sharing food and utensils or eating with a person with HBV**
   A. True
   B. False
   C. Don’t know
18. **Use condom**
   A. True

B. False
C. Don’t know

19. What is the best HBV prevention for children whose mother is a hepatitis B carrier or has chronic hepatitis B?

A. Administer the hepatitis B Vaccine
B. Administer HBIG shot
C. Administer HBV Vaccine within first 24 hours of birth
D. Administer the combination of HBIG shot and three doses of VGB Vaccine
E. Don’t know

20. Are you confident in consulting patients about preventions for HBV?

A. Yes
B. No
C. Not sure / Don’t know

21. In your opinion, who needs to be vaccinated to prevent hepatitis B infection? (*Please check ONE)*

A. All healthy and stable newborns
B. Family members of someone who has HBV
C. Sex partner of persons with HBV
D. Healthcare workers without immunity
E. All of the above

22. Do you think the hepatitis B vaccine is safe (*Please check ONE*):

A. Very safe
B. Maybe safe
C. Not very safe

23. When would you give a healthy and stable baby the first dose of hepatitis B vaccine? (*Please check ONE*)

A. Within the first 24 hours of birth
B. 1- 7 days old
C. 1 month old

24. What should a pregnant woman, who has hepatitis B, do to protect the newborn from becoming infected? (Please check ONE)

A. Administer hepatitis B vaccine to the pregnant woman
B. Administer the first dose of hepatitis B vaccine and the HBIG shot within 12 hours of birth then complete the vaccine series
C. Administer the first dose of hepatitis B vaccine and the HBIG shot after 48 hours of birth then complete the vaccine series

25. Do you feel confident in ordering HBV Vaccination for newborns?

A. Yes
B. No
C. Not sure / Don’t Know

26. Have you been tested for HBV before?

A. Yes
B. No -> Go to question 27

26B. What was the result of the test?

A. Positive for immunity to hepatitis B
B. Positive for chronic hepatitis B

C. Negative for immunity and chronic hepatitis B
D. Don’t remember / Don’t know

27. Have you been vaccinated against HBV?

A. Yes
B. No
C. Don’t remember / Don’t Know

28. Does the University of Medicine where you are studying require medical students to get vaccinated against HBV prior to to internship at hospital?

A. Yes
B. No
C. Not sure / Don’t Know

29. Does the University of Medicine where you are studying require medical students to get tested for HBV prior to to internship at hospital?

A. Yes
B. No
C. Not sure / Don’t Know

Question 30-34: As a medical student, which of the followings do you think can help healthcare workers to prevent infection from needlestick injury?

30. Wash hands with soap or disinfectant after each clinical procedure

A. True
B. False
C. Don’t know

31. Recap needle with two hands after use and discard immediately in a sharp-proof container

A. True
B. False
C. Don’t know

32. Do not recap needle and discard immediately in a sharp-proof container

A. True
B. False
C. Don’t know

33. Are there sharp-proof containers at your clinic for disposing needles and sharp objects? (*Please check ONE*):

A. Always
B. At some places
C. Not available

34. Do you wear glove when administrating injection to patients? (*Please check ONE*):

A. Always
B. Sometimes
C. Never

DIAGNOSIS ANS MANAGEMENT FOR HBV PATIENTS

Question 35-38: In your opinion, which of the following patient groups would you order hepatitis B screening test even if they have normal level of AST / ALT in liver, or don’t have hepatic symptoms?

35. Pregnant women

A. Yes
B. No
C. Don’t know

36. Persons infected with HIV

A. Yes
B. No
C. Don’t know

37. Men who have sex with men (MSM)

A. Yes
B. No
C. Don’t know

38. Family members of hepatitis B patients

A. Yes
B. No
C. Don’t know

39. Which single test would you order to confirm that a patient has chronic hepatitis B? (Please check ONE):

A. HBsAg
B. Anti-HBs
C. Anti-HBc
D. HBeAg

40. Which single test would you order to know if a patient has immunity to hepatitis B? (Please check ONE):

A. HBsAg
B. Anti-HBs
C. Anti-HBc
D. Anti-HBe
E. HBeAg

41. Have you encountered any patient with chronic hepatitis B infection?

A. Yes
B. No

42. In your opinion, what is the symptom most patients with chronic hepatitis B present? (Please check ONE):

A. Headache and fatigue
B. Nausea or vomiting
C. Loss of appetite
D. Jaundice
E. All of the above
F. None, there are usually no symptoms

43. Which measurement in people with positive HBsAg show the need to order for treatment? (*Please check all that apply*):

A. ALT level
B. HBV DNA
C. Evaluation of liver cirrhosis
D. The combination of the three above

44. Which of following statements is true about HBV treatment? (*Please check all that apply*):

A. HBV is curable
B. There is no cure, but there are medications effective to manage and control the disease
C. There is no treatment available, but there are herbal medicine that can help to slower the disease progression.

Question 45-48: What are goals of chronic HBV Treatment?

45. Sustain inhibition of HBV replication

A. True
B. False
C. Don't know

46. Improve quality of life and prevent progression to liver cirrhosis and liver cancer:

A. True
B. False
C. Don't know

47. Prevent spread of HBV infection in community, including infection from mother to child at birth:

A. True
B. False
C. Don't know

48. Prevent outbreak of HBV:

A. True
B. False
C. Don't know

Question 49-51: In your opinion, what are the rules in HBV treatment?

49. First line drugs are nucleot(s)ide analogues (NAs)

A. True
B. False
C. Don't know

50. Treatment of chronic HBV with NAs is long term, or can be for a life time

A. True
B. False
C. Don't know

51. Patients need to follow / obey the treatment process

A. True
B. False
C. Don't know

52. Do you think that all patients with chronic HBV need to be treated? (*Please check ONE*):

A. Yes, all patients with chronic HBV should receive treatment as soon as possible
B. Only patients with active liver damage or cirrhosis need to be treated
C. There is no need to treat HBV because there is no cure yet

53. Which of the followings is correct about monitoring HBV patients? (*Please check ONE*):

A. Only patients with symptoms need to be regularly monitored and screened
B. Only patients who are on HBV treatment need to be regularly monitored and screened
C. All patients with HBV need to be regularly monitored and screened, regardless of treatment indication

54. **Without proper monitoring and treatment, what is the chance a patients would die of complications of chronic hepatitis B? (*Please check ONE*):**
    A. Less than 5%
    B. 5-10%
    C. 15-25%
    D. >30-40%%
    E. Over 40%
55. **Are you confident in ordering the tests to monitor patients with chronic HBV?**
    A. Yes
    B. No
    C. Don't know / Not sure
56. **Are you confident in giving prescription to patients with chronic HBV?**
    A. Yes
    B. No
    C. Don't know / Not sure
57. **In your opinion, when should newborns from mothers who have positive HBsAg be evaluated / screened for their possibility of having HBV infection?**
    A. Right after birth
    B. 6 months
    C. 12 months
    D. 24 months
    E. Don't know
58. **What test should be done to diagnose patients with chronic HBV infection? *(please check ONE)***
    A. HBsAg
    B. Anti-HBs
    C. Anti-HBc
    D. HBeAg
59. **What test can show that a patient has immunity against HBV? *(please check ONE)***
    A. HBsAg
    B. Anti-HBs
    C. Anti-HBc
    D. Anti-HBe
    E. HBeAg
60. **Are you confident in ordering diagnosis test for patients with chronic HBV?**
    A. Yes
    B. No
    C. Don't know / Note sure
61. **Would you have any concern having casual contact or working together with a chronic HBV patients in the same office?**
    A. Yes
    B. No
    C. Somewhat concern
62. **Would you have any concern eating with (sharing food or utensils) with a chronic HBV patient?**
    A. Yes

B. No
C. Somewhat concern

MEDICAL STUDENT SURVEY

Answer to the questionnaire			
**Question Number**	**Answer**	**Question Number**	**Answer**
Q4	C	Q33	Always
Q5	A	Q34	Always
Q6	A	Q35	Yes
Q7	E	Q36	Yes
Q8	False	Q37	Yes
Q9	True	Q38	Yes
Q10	True	Q39	A
Q11	False	Q40	B
Q12	True	Q41	Yes
Q13	False	Q42	F
Q14	False	Q43	D
Q15	True	Q44	B
Q16	True	Q45	True
Q17	False	Q46	True
Q18	True	Q47	True
Q19	D	Q48	True
Q20	Yes	Q49	True
Q21	E	Q50	True

Q22	A	Q51	True
Q23	A	Q52	B
Q24	C	Q53	C
Q25	Yes	Q54	C
Q26		Q55	Yes
Q27		Q56	Yes
Q28	Yes	Q57	C
Q29	Yes	Q58	A
Q30	False	Q59	B
Q31	False	Q60	Yes
Q32	True	Q61	No
Q33	Always	Q62	No
